# Motor Performance is Impaired Following Vestibular Stimulation in Ageing Mice

**DOI:** 10.3389/fnagi.2016.00012

**Published:** 2016-02-03

**Authors:** Victoria W. K. Tung, Thomas J. Burton, Stephanie L. Quail, Miranda A. Mathews, Aaron J. Camp

**Affiliations:** ^1^Discipline of Biomedical Science, The University of SydneySydney, NSW, Australia; ^2^The Bosch Institute Animal Behavioural Facility, The University of SydneySydney, NSW, Australia; ^3^Discipline of Physiology, The University of SydneySydney, NSW, Australia; ^4^Brain and Mind Centre, The University of SydneySydney, NSW, Australia

**Keywords:** ageing, balance, vestibular, vestibular stimulus, motor coordination, vestibular hair cell

## Abstract

Balance and maintaining postural equilibrium are important during stationary and dynamic movements to prevent falls, particularly in older adults. While our sense of balance is influenced by vestibular, proprioceptive, and visual information, this study focuses primarily on the vestibular component and its age-related effects on balance. C57Bl/6J mice of ages 1, 5–6, 8–9 and 27–28 months were tested using a combination of standard (such as grip strength and rotarod) and newly-developed behavioral tests (including balance beam and walking trajectory tests with a vestibular stimulus). In the current study, we confirm a decline in fore-limb grip strength and gross motor coordination as age increases. We also show that a vestibular stimulus of low frequency (2–3 Hz) and duration can lead to age-dependent changes in balance beam performance, which was evident by increases in latency to begin walking on the beam as well as the number of times hind-feet slip (FS) from the beam. Furthermore, aged mice (27–28 months) that received continuous access to a running wheel for 4 weeks did not improve when retested. Mice of ages 1, 10, 13 and 27–28 months were also tested for changes in walking trajectory as a result of the vestibular stimulus. While no linear relationship was observed between the changes in trajectory and age, 1-month-old mice were considerably less affected than mice of ages 10, 13 and 27–28 months. Conclusion: this study confirms there are age-related declines in grip strength and gross motor coordination. We also demonstrate age-dependent changes to finer motor abilities as a result of a low frequency and duration vestibular stimulus. These changes showed that while the ability to perform the balance beam task remained intact across all ages tested, behavioral changes in task performance were observed.

## Introduction

It is well documented that advancing age is a significant risk factor for falls. Loss of balance and subsequent falls often lead to deleterious effects including repeated falls, anxiety, admission into high care facilities such as nursing homes and hospitals, and in some cases death in older adults (Rubenstein, 2006). Evidence shows that the majority of people at risk of experiencing falls also have symptoms of impairment when assessed using common tests of vestibular function such as caloric and Sinusoidal Harmonic Acceleration, and the Vertigo Symptom Scale questionnaire (Pothula et al., 2004; Jacobson et al., 2008). Vestibular impairment has been reported in subjects as young as 40 years of age and becomes more common with increasing age (Agrawal et al., 2009), yet the changes associated with the ageing vestibular system remain poorly understood.

The vestibular system comprises two main components: a peripheral component responsible for detecting accelerations of the head, and a central component that relays information from the periphery to central nervous system structures. These structures initiate reflexive responses to changes in head and body position and include the vestibulo-ocular (VOR), vestibulo-collic (VCR) and vestibulo-spinal reflexes (VSR). The reflexes can be used in the diagnosis of vestibular impairment (Halmagyi and Curthoys, 1988; Colebatch et al., 1994), and as such, age-related decline in balance function has been observed as a deficit in reflex activity. For example, reductions in VOR gain occur at high amplitude stimulation (Baloh et al., 1993) and decreased amplitudes in click-evoked VCR responses in older adults (Welgampola and Colebatch, 2001). While the VOR appears relatively stable in mice, a small decrease in VOR gain may occur during ageing (Stahl, 2004). Nonetheless, the underlying cause of these deficits is not clear and may manifest from alterations at any stage of vestibular processing from the sensory hair cells in the periphery to the output motor neurons.

Many studies on the peripheral vestibular system have been carried out using a range of animal species including the frog (Straka et al., 1997; Martini et al., 2007), turtle (Brichta et al., 2002), gerbil (Meredith and Rennie, 2015) and mouse (Lee et al., 2005). Despite information learned from this work, there is little understanding of how the properties of central and peripheral structures vary, and subsequently influence behavior during the process of natural ageing. Most age-related studies have focused on characterizing the changes that occur in the motor and balance performance of rodents (Wallace et al., 1980; Ingram et al., 1981; Gage et al., 1984). Indeed, results from studies using the common rotarod test suggest that age-related deficits in motor coordination begin as early as 4 months of age in mice. C57BL/6 mice showed improvements in motor coordination between juvenile mice at 1 month of age and adult mice at 3 months of age, before declining when tested at 7 and 13 months (Serradj and Jamon, 2007). Further, Fahlstrom et al. (2011) showed that 3 and 8-month-old mice display better motor coordination than 28-month-old mice—the mean life expectancy of laboratory mice (Fahlstrom et al., 2011; The Jackson Laboratory, 2014). The use of an elevated balance beam apparatus has also revealed age-related deficits in motor coordination and balance in rodents. C57BL/6 mice tested at 4 months of age performed better than those tested at 18, and 24 months (Ingram et al., 1981). This was consistent with the study conducted by Fahlstrom et al. (2011) who reported that motor performance declined between the ages of 3 and 28 months, with deficits most evident at 28 months of age. Studies using rats have also reported convincing age-related deficits in motor coordination on the balance beam with some aged subjects unable to complete the task (Wallace et al., 1980; Gage et al., 1984).

While it is likely that the changes described above result from a combination of vestibular, visual or proprioceptive alterations, the direct contribution of the vestibular system to balance impairment during ageing remains uncertain. Here, we confirm that both grip strength and gross motor performance decline with advancing age. We also demonstrate that a low frequency, short duration rotatory vestibular stimulus is sufficient to reveal age-related changes to balance performance. Finally, we show that a short intervention (access to a running wheel for 4 weeks as a form of voluntary exercise) is not sufficient to mitigate these age-related changes.

## Materials and Methods

### Animals

All procedures outlined were approved by the Animal Ethics Committee at The University of Sydney. C57BL/6J mice (ARC Perth, WA, Australia) between 1 and 29 months were tested on the behavioral tasks described below. All mice were held at The University of Sydney Bosch Rodent Facility on a 12 h light/dark cycle with access to food and water *ad libitum*. Prior to tests, mice were brought into the testing room for a 10 min acclimatization period.

### Assessment of Balance and Motor Performance

Mice were tested using a battery of balance and motor performance tasks as described below.

#### Grip Strength

The forelimb grip strength of mice was measured using a Digital Force Gauge (Chatillon, USA). Mice were held by the base of the tail close to the horizontal bar (attached to the force gauge) to allow them to reach and grab onto the bar with their forelimbs. Mice were then positioned so that their body was horizontal and in line with the bar. They were then pulled horizontally away from the bar by the tail until their grip was released. The tension was measured and defined as grip strength. Mice were given 1 min inter-trial intervals (ITI) during which they were returned to their cages with access to food and water. This procedure was repeated for a total of five trials for each mouse (with the median three values used for analysis). All grip strength values acquired were normalized for body weight.

#### Rotarod

**Figure 1:**
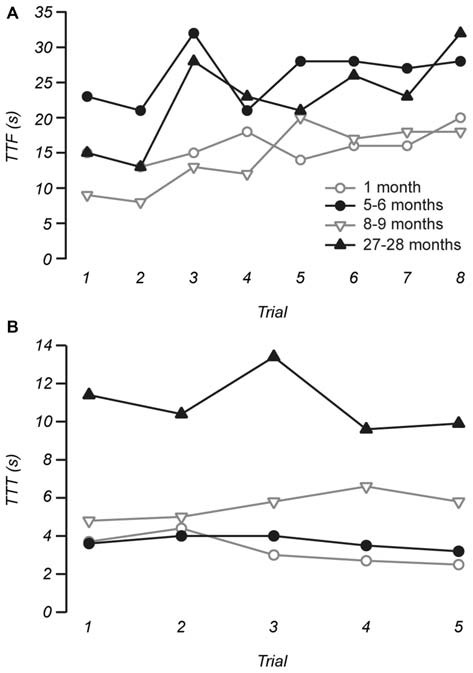
**Example measurements of time to fall (TTF) from the rotarod and time to traverse (TTT) the balance beam. (A)** The stability of TTF measurements was assessed by plotting TTF as a function of individual trials for each individual mouse. Across our sample and for the four individuals shown trials 1 to 3 on the rotarod were considered as training trials, while the stable measurements of the remaining five trials were used for data analysis. **(B)** Measurements of TTT on the balance beam were stable across trials, with trials 1 to 4 used as practice and measurements made from trial 5 were used to represent performance prior to the vestibular stimulus.

Motor performance was assessed using the protocol described in Tung et al. (2014). Briefly, gross motor control was measured using the rotarod (IITC Life Science, CA, USA). For this test, each mouse was placed on a cylindrical dowel (69.5 mm in diameter) raised 27 cm above the floor of a landing platform. Mice were placed on the dowels for 5 min to allow them to acclimatize to the test apparatus. Once initiated the cylindrical dowels began rotating and accelerated from 5 rpm to a final speed of 44 rpm over 60 s. During this time, mice were required to walk in a forward direction on the rotating dowels for as long as possible. When the mice were no longer able to walk on the rotating dowels, they fell onto the landing platform below. This triggered the end of the trial for an animal and measurements of time to fall (TTF) were collected. Passive rotations where mice clung to, and consequently rotated with the dowel were also used to define the end of the trial. Mice were then returned to their cages with access to food and water for 10 min. This procedure was repeated for a total of eight trials, with the first three trials used for training and subsequent trials used for data analysis. Figure 1A shows the stabilization of performance as measured by TTF for four individual mice of different ages as a function of successive trials.

#### Balance Beam

More subtle motor coordination and balance was assessed using a modified balance beam test (Tung et al., 2014). After initial training, mice were given four practice trials where they were required to traverse 60 cm of an elevated round beam (14 mm in diameter) in order to reach the shelter of a goal box. This was followed by a 5th trial, which was used for data analysis. The vestibular system was then challenged with a vestibular stimulus where mice were placed in an opaque, low contrast chamber that rotated, accelerating from rest to a maximum speed of 2–3 Hz for 20 s. The maximum amplitude of the stimulus was 0.60–1.36 g. Immediately after the vestibular stimulus, mice were retested on the balance beam. Mice were given 1 min ITI during which they remained inside the darkened goal box positioned at the end of the balance beam. All trials conducted were video recorded at 30 fps for subsequent analysis. Measurements recorded for analysis include time to start (TTS) traversing after being placed on the beam, time taken to traverse the beam (TTT), and the number of times the hind-feet slip (FS) from the beam while traversing. Figure 1B shows that TTT was remarkably consistent between trials and across ages.

For mice that fell or were unable to perform the task, the highest value for TTT and FS recorded for the age group was assigned. Similarly, mice unable to perform the task post-vestibular stimulus were assigned the highest post-stimulus value recorded for that age group. If mice fell beyond the starting line on the beam, TTS was recorded as normal, however if the mouse fell immediately after being placed on the beam or before reaching the start line, the highest value of TTS recorded for that age group was allocated to that trial.

To mitigate the effect of visual input during the vestibular stimulus the interior surface of the chamber was lined with black contact paper. To lessen the influence of normal gait width on balance beam performance, 1-month-old mice were also tested on a smaller 9.5 mm diameter beam.

#### Walking Trajectory

Mice were trained to walk diagonally from one corner of a clear Perspex box (dimensions: 42.5 × 29.5 × 23 cm) to the opposite corner where a darkened goal box was located. The floor of the arena was lined with tracing paper. Training was carried out in a similar manner to that used for balance beam experiments, where mice were progressively placed further away from the goal box and towards the starting location until they were able to walk 30 cm unassisted from the starting position to the goal box. Using a cotton tip, petroleum jelly (Vaseline) was applied to the soles of the mouse’s hind-feet. Mice were then placed at the starting position so that they could walk to the goal box. This was repeated in instances where mice did not walk directly to the goal box or required encouragement by prodding, and was repeated until mice performed the task unassisted. The unassisted trial was used for analysis. Mice then underwent the vestibular stimulus (as above) and were immediately placed back at the starting position to perform the task. During the testing of 1-month-old mice, an additional light source was used to encourage mice to walk towards the goal box.

For analysis, graphite powder (Pressol) was used to color the petroleum jelly residue on the tracing paper (Lee et al., 2012). Photos of the tracing paper were analyzed using ImageJ version 1.49d (NIH). All photos were scaled along the vertical axis and converted into grayscale images. A pixel intensity threshold of 100 was then applied to create a binary image. An ellipse was then fitted to the walking path of mice and the length of the minor axis was used as a measure of deviation from the most direct walking route between the start position and entry into the goal box.

#### Running Wheel

To assess the impact of short-duration voluntary exercise on the motor and balance performance of aged mice, a running wheel (Med Associates Inc., VT, USA) was added to each cage of 27–28-month-old mice. The running wheel remained in the cages for 4 weeks and were wirelessly connected to a PC in order to monitor running wheel usage in each cage of mice. After this period, mice were retested on all behavioral tasks.

#### Data Analysis

IBM SPSS Statistics software (Version 21) was used for statistical analysis. Unless otherwise stated, analysis was conducted using either two-tailed Student’s *t*-test or ANOVA, or their non-parametric equivalent, and significance was set at *p* < 0.05. Either Tukey or Bonferroni *post hoc* tests were used for multiple comparisons. Values are reported as mean ± *SD*.

## Results

### Grip Strength and Gross Motor Performance Declines with Age

**Figure 2:**
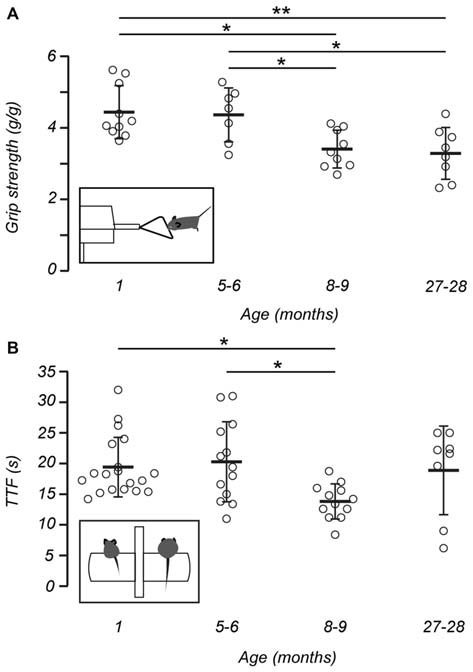
**Gross motor performance and grip strength declines with age. (A)** The grip strength of mice, normalized for weight, declined with age. *Post hoc* analysis showed 1 (*n* = 10) and 5–6-month-old mice (*n* = 7) were stronger than 8–9-month-old mice (*n* = 9) and 27–28-month-old mice (*n* = 8). Inset shows a representation of a mouse undergoing the grip strength test. **(B)** Measurements of time to fall (TTF) decline with increasing age. *Post hoc* analysis showed differences in TTF between 1 (*n* = 19) and 8–9-month-old mice (*n* = 12), and 5–6 (*n* = 13) and 8–9-month-old mice. No difference was observed between 27–28-month-old mice (*n* = 8) and other age groups. Inset shows a schematic of a 1-month-old mouse (left) and 8–9 –month-old mouse (right) on the rotarod apparatus in preparation for the rotarod test. Open circles represent the mean recorded for individual mice, horizontal bars indicate the mean for each age group, error bars = *SD*, *p < 0.05, **p < 0.01.

Previous work has shown that the grip strength of mice deteriorates with age (Fahlstrom et al., 2011). As expected we also observed that fore-limb grip strength of mice declined with age when measurements were normalized for weight. (One-way ANOVA, *F*_(3,30)_ = 6.727, *p* = 0.001). A *post hoc* test showed that both 1 (4.44 ± 0.74 g/g) and 5–6-month-old mice (4.36 ± 0.75 g/g) were stronger than 8–9-month-old mice (3.41 ± 0.53 g/g, *p*s < 0.05) and 27–28-month-old mice (3.29 ± 0.73 g/g, *p* < 0.01 and *p* < 0.05, respectively; Figure 2A). However, no difference was observed between 1 and 5–6-month-old mice.

Mice aged between 1 and 27–28 months were assessed for gross motor performance and coordination using an accelerating rotarod test. The performance of each mouse was measured as the TTF from the rotating drum onto a landing platform. Based on this test, gross motor performance declined with increasing age (Kruskal-Wallis H, χ^2^ (3) 11.431, *p* = 0.01). When individual age groups were compared, a number of differences were observed. First, *post hoc* analysis showed that 8–9-month-old mice (13.81 ± 2.86 s) displayed shorter TTF when compared with 1-month-old mice (19.42 ± 4.86 s; *p* < 0.05) and 5–6-month-old mice (20.28 ± 6.54 s, *p* < 0.05; Figure 2B). Interestingly, the motor performance of 27–28-month-old mice (18.88 ± 7.22 s) did not differ from any other age group.

### Balance and Motor Coordination Declines in Older Animals After Vestibular Stimulation

Mice of ages 1, 5–6, 8–9, and 27–28 months old were tested on the balance beam apparatus before and after a rotatory vestibular stimulus. In some instances, mice were unable to perform the task and fell from the balance beam apparatus, particularly in the 27–28 month age group where three of the eight mice fell in the trial prior to the vestibular stimulus and four fell in the trial after the vestibular stimulus. One 8–9-month-old mouse also fell after undergoing the vestibular stimulus, however none of the 1 or 5–6-month-old mice fell.

Three different measures were used to assess changes in balance beam performance across all age groups: TTS, TTT and number of foot slips (FS). These measurements were recorded both before and after the vestibular stimulus (Table 1). Baseline measurements of TTS did not change with age (Kruskal-Wallis H, χ^2^ (3) 5.948, *p* = 0.114). In contrast, baseline measurements of TTT (Kruskal-Wallis H, χ^2^ (3) 19.652, *p* < 0.001) and FS (Kruskal-Wallis H, χ^2^ (3) 15.120, *p* = 0.002) both showed increases with age.

**Table 1:** **Balance beam measurements observed for each group before and after vestibular stimulation**.

Balance beam measurements	1 month (*n* = 9)	5–6 months (*n* = 7)	8–9 months (*n* = 7)	27–28 months (*n* = 8)
TTT (s)
Before	0 ± 0	0 ± 0	0 ± 0	0.55 ± 1.02
After	0.04 ± 0.13	0.93 ± 2.09	2.81 ± 2.05	5.1 ± 5.01
TTT (s)
Before***	3.37 ± 0.89	3.51 ± 0.68	6.03 ± 3.59	12.2 ± 3.66
After	3.77 ± 0.67	4.8 ± 1.11	6.46 ± 2.05	12.53 ± 2.59
FS
Before**	0.22 ± 0.44	1.57 ± 1.99	1.71 ± 2.06	28.75 ± 14.57
After	0.89 ± 1.27	1.29 ± 2.36	1.57 ± 1.81	35.13 ± 19.18

Balance beam measurements taken before vestibular stimulation show times to traverse (TTT) and the number of foot slips (FS) observed increase with age but no changes evident for time to start traversing (TTS). **p < 0.01, ***p < 0.001. Values expressed as mean ± SD.

**Figure 3:**
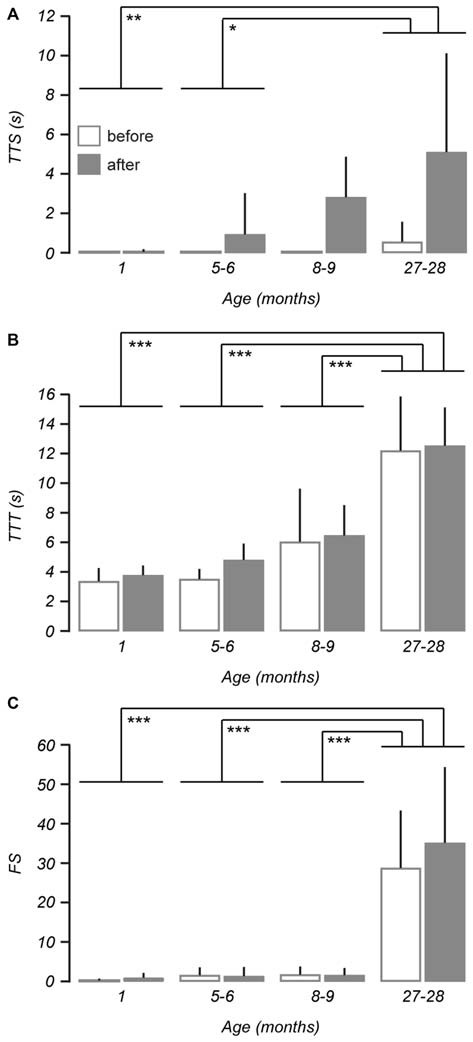
**Balance and motor performance is impaired in older mice after vestibular stimulation.** Age-related changes were assessed using three different measures of balance beam performance across 1 (*n* = 9), 5–6 (*n* = 7), 8–9 (*n* = 7) and 27–28-month-old mice (*n* = 8). **(A)** The effect of the vestibular stimulus on time to start (TTS) walking was increased with age such that 27–28-month-old mice were more affected than 1-month-old and 5–6-month-old mice. **(B)** Changes observed in measurements of time to traverse (TTT) as a result of the vestibular stimulus were not age-dependent, however differences were apparent between 27–28-month-old mice and other age groups. **(C)** An age-related effect of the vestibular stimulus was also identified by the number of foot slips (FS). Error bars = *SD*, *p < 0.05, **p < 0.01, ***p ≤ 0.001.

Following the vestibular stimulus an age-related increase in measurements of TTS was observed (repeated measures ANOVA, *F*_(3,27)_ = 3.573, *p* = 0.027), with the *post hoc* test showing 1 (*p* < 0.01) and 5–6-month-old (*p* < 0.05) mice were affected by the stimulus differently to 27–28-month-old mice (Figure 3A). There was no age-dependent effect of the vestibular stimulus on measurements of TTT (Figure 3B), however an age-related difference was apparent in the incidence of FS (repeated measures ANOVA, *F*_(3,27)_ = 4.572, *p* = 0.010; Figure 3C). *Post hoc* tests revealed greater increases in TTT and FS for 27–28-month-old mice when compared to 1 (*p*s < 0.001), 5–6 (*p*s < 0.001), and 8–9-month-old mice (*p*s < 0.001).

Due to their age, 1-month-old mice have comparably smaller gait widths than the older mice. Therefore, to assess whether a smaller gait width to beam diameter ratio resulted in better balance beam performance in younger mice, 1-month-old mice (*n* = 5) were tested on a smaller (9.5 mm diameter) beam using the same balance beam protocol and compared with 1-month-old control mice reported above (*n* = 9). No differences in baseline measurements were observed due to the different beam diameters. Furthermore, the different beam diameters did not result in changes in balance beam measures following the vestibular stimulus.

To determine whether visual input during the vestibular stimulus influenced balance beam outcomes (e.g., via the VOR), black contact paper was used to line the interior surface of the vestibular stimulus chamber. When comparing 8–9-month-old mice with (*n* = 6) and without (*n* = 6) the darkened vestibular chamber, no differences were shown for all balance beam measures. Additionally, when 1 (*n* = 6) and 8–9-month-old mice (*n* = 6) were tested using the darkened chamber, no age-related changes were evident, suggesting the results described above are independent of visually-mediated effects.

### Age-Related Changes in Walking Trajectory Due to Rotatory Vestibular Stimulus in Older Mice are Non-Linear

**Figure 4:**
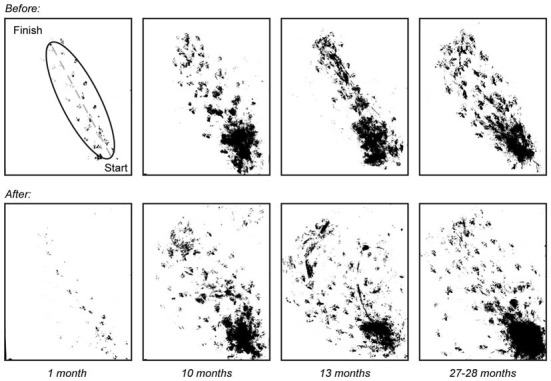
**Walking trajectory before and after vestibular stimulus.** Photos of walking patterns obtained for mice of ages 1 (*n* = 3), 10 (*n* = 7), 13 (*n* = 6) and 27–28 (*n* = 8) months were overlaid before and after the vestibular stimulus. An ellipse was fitted to the walking paths of each mouse and the length of the minor axis was measured and used to quantify changes in walking trajectory with age.

Walking trajectory was assessed in mice of ages 1, 10, 13, and 27–28 months. For this test, mice traversed from the designated start position to the goal box. Deviations from the most direct route were quantified by the length of the minor axis of an ellipse fitted to the walking path of each mouse. An age-related increase in minor axis length was observed from baseline measurements of walking trajectory (one-way ANOVA, *p* = 0.001), with 1-month-old mice having much smaller minor axis length than 10 (*p* = 0.001), 13 (*p* < 0.01), and 27–28-month-old mice (*p* < 0.01, Figure 4).

**Figure 5:**
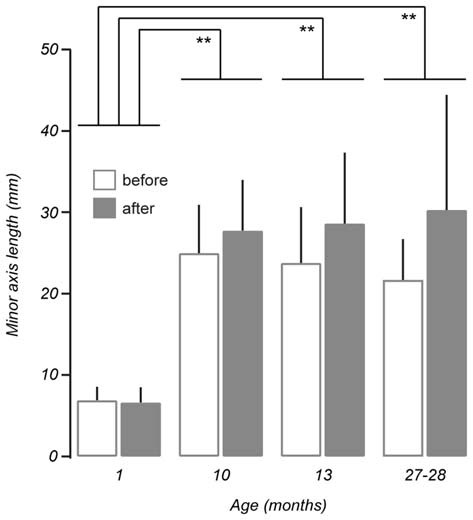
**Comparison of mean minor axis length before and after vestibular stimulation for all age groups tested.** No linear interaction was found between the effect of the vestibular stimulus and ageing (*p* > 0.05, repeated measures ANOVA). *Post hoc* comparisons showed non-linear, age-related differences in minor axis length between 1-month-old mice (*n* = 3) and 10 (*n* = 7, *p* < 0.01), 13 (*n* = 6, *p* < 0.01), and 27–28-month-old mice (*n* = 8, *p* < 0.01). Error bars = *SD*, **p < 0.01.

Measurements taken after the vestibular stimulus showed there was a non-linear relationship between the effect of the stimulus on minor axis length and age such that minor axis length did not increase as a function of age (*p* > 0.05). Instead, *post hoc* tests revealed that changes in minor axis length in 1-month-old mice as a result of undergoing the vestibular stimulus (before: 6.90 ± 1.66 mm vs. after: 6.64 ± 1.85 mm), were significantly different from those observed in 10 (before: 24.98 ± 5.93 mm vs. after: 27.74 ± 6.23 mm, *p* < 0.01), 13 (before: 23.79 ± 6.85 mm vs. after: 28.61 ± 8.71 mm, *p* < 0.01), and 27–28-month-old mice (before: 21.70 ± 5.00 mm vs. 30.28 ± 14.13 mm, *p* < 0.01; Figure 5). No differences were observed between 10, 13, and 27–28-month-old mice. This finding suggests that that the vestibular-mediated impact of age on walking trajectory develops relatively early but subsequently reaches a plateau.

### 4-Week Running Wheel Intervention has Minimal Effect on Motor Behavior and Balance in Aged Mice

After initial testing of 27–28-month-old mice with the grip strength, rotarod, balance beam, and walking trajectory tests, running wheels were added to their cages. These running wheels were provided as a form of voluntary exercise and remained in the cages for a period of 4 weeks, at the end of which mice were retested. During the 4-week period with a running wheel, two mice were euthanized due to poor health (see “Discussion” Section) and no changes in grip strength or TTF were observed as a result of adding a running wheel into home cages.

During balance beam tests, three of the eight 27–28-month-old mice fell during the trial prior to the vestibular stimulus and four mice fell after the stimulus (as above). Two of these mice fell before and after the stimulus and were excluded from further testing due to health problems. At 29 months, after a running wheel was added to home cages for 4 weeks, three of the six mice fell in the trial prior to the stimulus. Two of these mice also fell after the vestibular stimulus, and had fallen during testing at 27–28 months of age. There were no changes in baseline measurements of TTS and TTT as result of the running wheel, though there was an increase in the number of FS observed at 29 months of age (46.67 ± 20.59****; Mann-Whitney *U* = 7, *p* = 0.029). The running wheel did not improve balance beam measures post-vestibular stimulus. There was also no improvement in walking trajectory observed as a result of adding a running wheel.

## Discussion

Here, we assessed the balance, motor coordination, walking trajectory and grip strength of mice and describe changes that occur across their lifespan. We also investigated their responses to a low frequency rotatory vestibular stimulus and whether these responses are altered as a result of ageing.

### Considerations on Mouse Age

In this study, mice from 1–29 months of age were tested. At 1 month of age, mice have been described as “juvenile” with abilities to swim, walk, and grasp objects (Fox, 1965). In the vestibular epithelium, the proportion of type I vestibular hair cells identified in the utricle of the mouse (Rüsch et al., 1998) is similar to those previously reported in the guinea pig (Lindeman, 1969), although afferent signaling remains immature without the low sensitivity irregular afferents identified in adult mice (Lasker et al., 2008). By 3 months of age mice are adults and are classified as middle age at 14 months of age (Lasker et al., 2008; Cheng et al., 2013). Mice that were tested at 27–28 months were retested at 29 months of age, except for two mice that were euthanized due to health problems. At 29 months of age mice are well past their reported life expectancy of 25.64 months for females and 26.79 months for males (The Jackson Laboratory, 2014). Some mice in this age group displayed signs of age-related disorders, including kyphosis and cloudy eyes. Fahlstrom et al. (2011) also reported changes in physical health in 29-month-old mice including a reduction in weight and the presence of gray fur. Although the number of mice exhibiting signs of physical ageing was not quantified in this study, it would not be surprising if they influenced motor performance and resulted in greater variability in test measures across the age group, as suggested by the standard deviations in balance beam measurements of TTS, TTT, and FS (Figure 3). A previous study by Cheng et al. (2013) has also noted greater variability in older mice when examining acetylcholine receptor area in endplates at neuromuscular junctions.

### Assessment of Strength and Motor Performance

Previous age-related studies on grip strength have led to varied results. Fahlstrom et al. (2011) reported a decrease in grip strength between ages 15 and 28 months in C57Bl/6 mice. In contrast, Ingram et al. (1981) reported no age-related changes in the same strain of mice between 4 and 24 months of age. We also assessed the forelimb grip strength of mice across our age range, and as, predicted observed a decrease in forelimb grip strength with age. This decrease in grip strength occurred mainly between ages 5–6 and 8–9 months. It is not clear where the discrepancy in grip strength results arises, although in the case of Fahlstrom et al. (2011) the method used to assess grip strength (suspension test) varied to that used here and in Ingram et al. (1981). Further, it is not clear how reductions in grip strength with advancing age influence subsequent measurements of motor performance. For this reason, we compared performance before and after a rotatory stimulus in the same animal, thus negating the impact of grip strength and other potential confounders such as weight, which have been shown to impact on rotarod performance (McFadyen et al., 2003).

The rotarod test is commonly used in the assessment of motor coordination to characterize disease models (Carter et al., 1999), determine the effects of pharmacological practises (Cartmell et al., 1991) and phenotype strain and/or genetic profiles (Ingram et al., 1981; Serradj and Jamon, 2007). Here, we used the rotarod to show that motor coordination declines during ageing, particularly beyond 8–9 months of age. The age-related decline in motor coordination reported in this study is consistent with Serradj and Jamon (2007), who observed a decrease from as early as 7 months. While we saw no differences in motor coordination between 27–28-month-old mice and other age groups, Fahlstrom et al. (2011) reported that 3 and 8-month-old mice performed better than 28-month-old mice. Interestingly, Ingram et al. (1981) showed no differences between 4, 18, and 24-month-old mice, which may suggest that the deleterious effects occur before 4 months of age and plateau. The precise timeline of age-related deterioration in motor performance was not assessed here, but our findings support the idea that age-related deterioration occurs in a non-linear fashion.

### Age-Related Changes in Balance Performance

Similar to previous work in mice (Ingram et al., 1981; Fahlstrom et al., 2011), our balance beam data show that gross balance performance deteriorates during ageing. We show that TTT and the number of FS were significantly higher in 27–28-month-old mice. Ingram et al. (1981) showed an age-related decline in their evaluation of balance ability in mice ranging from 4–24 months old with no differences observed between ages 18 and 24 months. Further, Fahlstrom et al. (2011) showed that declines in walking speed were evident by 15 months and that the number of FS observed also increased at 28 months. In addition, approximately half the mice at 28 months of age did not successfully perform the task in the study by Fahlstrom et al. (2011), consistent with our observation that three of the eight 27–28-month-old mice fell from the beam. In our analysis we also observed a different strategy for traversing the beam between the young and old mice. Some 27–28-month-old mice traversed the balance beam with their hind feet grasping the sides of the beam. Siegel (1970) observed that mice with no tails also adopted this technique, presumably to compensate for compromised ability to maintain balance on the beam. In support of this, previous studies on rats suggest that older animals retain the ability to recover from brain injuries such as ischemia and seizure (Gray et al., 2002; Jin et al., 2004) as reviewed in Popa-Wagner et al. (2011), and in addition, age-related differences in gene expression have been identified post-ischemia (Buga et al., 2012) and may account for this compensation.

### Vestibular Stimulation

A key question posed by our study was the contribution of the vestibular system to the age-related changes in balance performance observed. We used a novel approach to stimulate hair cells in the semicircular canals of the peripheral vestibular labyrinth and tested for age-related differences in subsequent balance beam performance. The vestibular stimulus used was of low frequency and short duration, with the aim of stimulating the hair cells of the semicircular canals (predominately the horizontal semicircular canal). Other studies have used centrifuges to induce motion sickness in rats, and have also measured consumption of either saccharin (Xiaocheng et al., 2012) or kaolin (Yu et al., 2007) to measure the level of motion sickness experienced by the rats. Here, no attempt was made to quantify the degree of dizziness experienced by the mice, since it would delay testing on the behavioral apparatus following vestibular stimulation and potentially attenuate impact of the stimulus.

Using this approach, our results demonstrate that with increasing age, both TTS and the incidence of FS increase as a result of vestibular stimulation, particularly in the 27–28-month-old age group. Since no age-dependent effect of the vestibular stimulus was observed in measurements of TTT, the results suggest that ageing alters the ability of mice to recover from vestibular hair cell stimulation, rather than the ability to perform the task. Additionally, our results also indicate this deterioration is likely to occur between ages 8–9 months and 27–28 months. A potential contributing factor to this reduced capacity to recover from hair cell stimulation is the decrease in VOR gain as suggested in the study by Stahl (2004). Furthermore, both a decrease in VOR gain and an increase in VOR latency have been observed in older adults in humans (Baloh et al., 1993; Tian et al., 2002).

One possibility that could account for the changes we observed was that the larger size and weight of the older mice may have shifted their center of mass beyond the width of the beam and artificially reduced their performance on the beam in comparison to younger mice where the center of mass remained within the dimensions of the beam. To account for this, 1-month-old mice were tested with a smaller 9.5 mm diameter beam (normalized to normal gait width). No differences were observed in the performance of young mice between the small and large beams. Further, stimulation of the vestibular system with the rotatory stimulus did not alter performance on the smaller beam. This suggests that deterioration in balance (with and without stimulation of hair cells) as age increases is not due to changes in gait width or weight which has the potential to hinder the ability for mice to grip onto the beam (Curzon et al., 2009). A previous study by Altun et al. (2007) showed that although there was a fall in weight measurements in aged rats, balance beam scores continued to deteriorate. A second possibility is that stimulation of the visual system during rotation, and the potential differences in visual performance between young and older mice may have influenced post-rotation balance beam measurements. To alleviate this, matte black paper lined the inside of the vestibular stimulus chamber in a subset of experiments providing limited contrast or motion signals. We observed no differences in balance beam measures with or without reduced contrast suggesting that age-related declines in balance beam performance post-vestibular stimulus are not visually mediated.

### Assessment of Walking Trajectory

For walking trajectory experiments, deviations from the most direct walking route between the start location and the goal box was measured. Overall statistical analysis showed that stimulation of the vestibular hair cells had a non-linear effect on walking trajectory across the ages tested, with *post hoc* multiple comparisons revealing differences between 1-month-old mice and 10, and 27–28-month-old mice. Importantly, some mice were observed to stay at the start position for a variable period after the vestibular stimulus and before beginning to walk towards the goal box. This delay, presumably used by mice to recover from the vestibular stimulus, was not quantified for these experiments, but likely represents the TTS measurement for the balance beam task. For this reason, the subsequent walking trajectories reported here may underestimate the impact of vestibular stimulation on walking trajectory. Finally, it should be noted that during initial walking trajectory experiments, a light source was placed behind the start location to encourage mice to walk towards the relatively darker goal box (Costall et al., 1989). Since minimal improvement in completing the walking trajectory task was observed, the light source was removed from subsequent experiments.

### Voluntary Exercise as an Intervention Against Age-Related Balance Decline

As a form of voluntary exercise, a running wheel was added to the home cages of aged (27–28-month-old) mice for 4 weeks. We observed an increase in the number of FS prior to the vestibular stimulus, and no improvements in performance after the vestibular stimulus that were attributable to the running wheels. A previous study has reported running wheels improve the motor coordination of younger mice. Mice housed with running wheels after weaning and tested at approximately 3 months of age scored better in the accelerating rotarod test than mice given a locked running wheel (Pietropaolo et al., 2006). Whether the effectiveness of the running wheel to improve motor performance is dependent on the duration mice had access to the running wheel, or the age at which it was provided to mice, remains unclear. Since more than one mouse was housed in the same cage, running wheel usage was not further analyzed to determine if changes in test measures were correlated to running wheel usage.

## Conclusion

Currently, there are few studies that characterize age-related changes in balance performance, and even fewer that investigate age-related changes to vestibular-mediated balance performance. This study aimed to investigate the changes that occur when the vestibular hair cells are stimulated and shows that age impacts on vestibular-mediated motor coordination and balance performance. This information provides the impetus to investigate age-related changes in vestibular physiology at the level of the vestibular periphery (i.e., the vestibular hair cells and primary afferents), and the central vestibular components that form the output of the balance system.

## Author Contributions

VWKT, TJB, SLQ, MAM, and AJC were involved in developing and conducting experiments, data analysis, and writing and/or editing of the manuscript.

## Conflict of Interest Statement

The authors declare that the research was conducted in the absence of any commercial or financial relationships that could be construed as a potential conflict of interest.
